# Kinetic Study of Acetone-Butanol-Ethanol Fermentation in Continuous Culture

**DOI:** 10.1371/journal.pone.0158243

**Published:** 2016-08-03

**Authors:** Edward A. Buehler, Ali Mesbah

**Affiliations:** Department of Chemical and Biomolecular Engineering, University of California, Berkeley, CA 94720, United States of America; Korea University, REPUBLIC OF KOREA

## Abstract

Acetone-butanol-ethanol (ABE) fermentation by clostridia has shown promise for industrial-scale production of biobutanol. However, the continuous ABE fermentation suffers from low product yield, titer, and productivity. Systems analysis of the continuous ABE fermentation will offer insights into its metabolic pathway as well as into optimal fermentation design and operation. For the ABE fermentation in continuous *Clostridium acetobutylicum* culture, this paper presents a kinetic model that includes the effects of key metabolic intermediates and enzymes as well as culture pH, product inhibition, and glucose inhibition. The kinetic model is used for elucidating the behavior of the ABE fermentation under the conditions that are most relevant to continuous cultures. To this end, dynamic sensitivity analysis is performed to systematically investigate the effects of culture conditions, reaction kinetics, and enzymes on the dynamics of the ABE production pathway. The analysis provides guidance for future metabolic engineering and fermentation optimization studies.

## Introduction

Recent concerns about depleting crude oil reserves, environmental impact of fossil fuels, and national security threats have prompted increased interest in development of alternative fuels [1]. Biobutanol derived from sustainable renewable resources such as lignocellulosic biomass has emerged as a promising renewable drop-in fuel [2–5]. In comparison with bioethanol, biobutanol has higher energy density and can be used in 100% blends, while being less hygroscopic that facilitates its transport via pipelines [2, 3, 6, 7].

Biobutanol can be produced by bacteria of genus *Clostridium* (*C*.) in a process known as the acetone-butanol-ethanol (ABE) fermentation. The ABE fermentation is a biphasic process that converts sugars into acids (acetate, butyrate) and solvents (acetone, butanol, ethanol). During the first phase, acidogenesis, the primary products are the acidic metabolites. As the metabolism shifts to solventogenesis, the acids are assimilated into the ABE solvents. While this metabolic shift is associated with changes in the extracellular pH and the onset of sporulation, its exact mechanism is not understood [8, 9]. Recent experiments have shown that enzyme regulation plays a key role in the phase shift [10]. The ABE fermentation is also dependent on various culture conditions such as pH [8, 11, 12], nutrient shortage [9, 13], product inhibition [14, 15], media composition [16–18], and redox state [19, 20]. The most prevalent Clostridium bacteria for the ABE fermentation are *C. acetobutylicum* and *C. beijerinckii*, though the more recent *C. saccharoperbutylacetonicum* N1-4 strain has garnered interest due to high butanol yields [7, 21].

The ABE fermentation by clostridia in batch cultures is a well-established industrial process that dates back to the early 1900s [2, 5, 22]. Initially discovered while searching for a process to produce synthetic rubber, the batch clostridial ABE fermentation eventually became the second-largest industrial bioprocess before falling out of favor in the 1950s due to the increasing worldwide oil supplies [23–25]. Because of low productivity (0.1–0.3 g/L/h) of the batch ABE fermentation due to long process downtime, the ABE fermentation in continuous cultures has garnered interest for large-scale production of biobutanol [26]. The continuous ABE fermentation offers several advantages over the batch fermentation—improved productivity due to less process downtime, integration with downstream units, and increased titer due to continuous product removal [2, 6, 27]. However, the continuous ABE fermentation exhibits poor long-term stability (resulting in washout) [13] that leads to low product titer, productivity, and yield [4, 7, 8, 25, 28]. To address the latter shortcomings of the continuous ABE fermentation, a great deal of work has been done on metabolic engineering of the ABE production pathway to improve butanol tolerance and selectivity of the *C*. bacteria [3, 29]. On the other hand, systems analysis of the continuous ABE production based on kinetic modeling of its metabolic pathway has received much less attention [7]. Systematic analysis of the fermentation kinetics in relation to the continuous culture conditions can provide guidance for metabolic engineering as well as fermentation design and optimization studies.

Kinetic modeling of fermentation processes aims at describing the time evolution of concentration of different metabolites by accounting for their rates of formation and consumption in the metabolic pathway. The early work on modeling of the ABE fermentation mainly involved developing stoichiometric models based on a flux balance of the system at steady state (e.g., [30, 31]). Stochiometric models lack the ability to describe the fermentation behavior under transients and, thus, have limited predictive capability when culture conditions change. Early attempts on modeling of the ABE fermentation also entailed development of several kinetic models that relied on a reduced version of the metabolic pathway with only a few key metabolites (e.g., [32–34]). The kinetic models were intended to describe the effects of culture conditions such as dilution rate, media composition, and pH on the ABE production (see [35] for a recent review on mathematical modeling of ABE fermentation).

More recent kinetic modeling work for the batch ABE fermentation has demonstrated the importance of the intermediates as well as the effects of enzyme regulation. Shinto et al. [36] reported a kinetic model that included multiple intermediates in the metabolic pathway of *C. saccharoperbutylacetonicum* N1-4, while accounting for product inhibition and metabolic response to glucose inhibition. Li et al. [37] extended the latter kinetic model by including butyryl-phosphate, an intermediate that can predict the shift from acidogenesis to solventogenesis, and the metabolic regulatory effects of transcriptional control. In [38], a metabolic model based on an extensive number of genes, reactions, and metabolites was presented to investigate the solventogenic stress response of *C. acetobutylicum*. The inclusion of a large number of metabolites and genes allowed for successful prediction of the switch from acidogenesis to solventogenesis and the effect of culture conditions. The most advanced kinetic model of the batch ABE fermentation is that of Liao et al. [39], which integrated modules for gene regulation, environmental cues, and metabolic reactions to develop a systems-level kinetic model. This novel modeling framework allowed for investigation into how conditions at both the cellular scale and in the culture affect the fermentation. Recent efforts have also been made to develop kinetic models of the continuous ABE fermentation with the aim of providing similar insights into how culture conditions affect the fermentation. Haus et al. [40] presented a kinetic model that considered some of the important enzymes in continuous culture and their pH dependence. The latter kinetic model was extended in [41–43] to include the role of cell population dynamics as well as other enzymes involved in the metabolic shift from acidogenesis to solventogenesis. These kinetic models, however, provide limited investigation into the effects of process and kinetic parameters on the systems-level fermentation. Furthermore, several intermediates in the kinetic models of [36, 37], shown to have significant effects on the batch fermentation dynamics, were omitted in the kinetic model of [40–43] for the continuous ABE fermentation.

The aim of this paper is to present a kinetic model for the ABE fermentation in continuous *C. acetobutylicum* that can be used for elucidating the behavior of the fermentation under culture conditions most relevant to continuous ABE production. The kinetic model mainly relies on the metabolic pathway presented in [36], and accounts for the effects of biomass changes, enzyme regulation, culture pH, product inhibition, and glucose inhibition. The intermediates are chosen such that the model can describe the key characteristics of the metabolic pathway while avoiding its overparametrization (due to selecting an excessive number of intermediates). Existing data from literature [44] are utilized to estimate the parameters of the kinetic model using the weighted least-squares parameter estimation method. After characterizing the uncertainty associated with the estimated parameters, the kinetic model is employed for systems analysis of the continuous ABE fermentation. Extensive sensitivity analysis is performed to systematically investigate the effect of various culture conditions (such as pH and dilution rate), reaction kinetics, and enzyme regulation on continuous ABE production in *C. acetobutylicum* culture.

## Methods

This section describes the metabolic pathway of *C. acetobutylicum* adopted for developing the kinetic model. The modeling framework and the parameter estimation method are discussed in this section.

### ABE pathway

**Fig 1:**
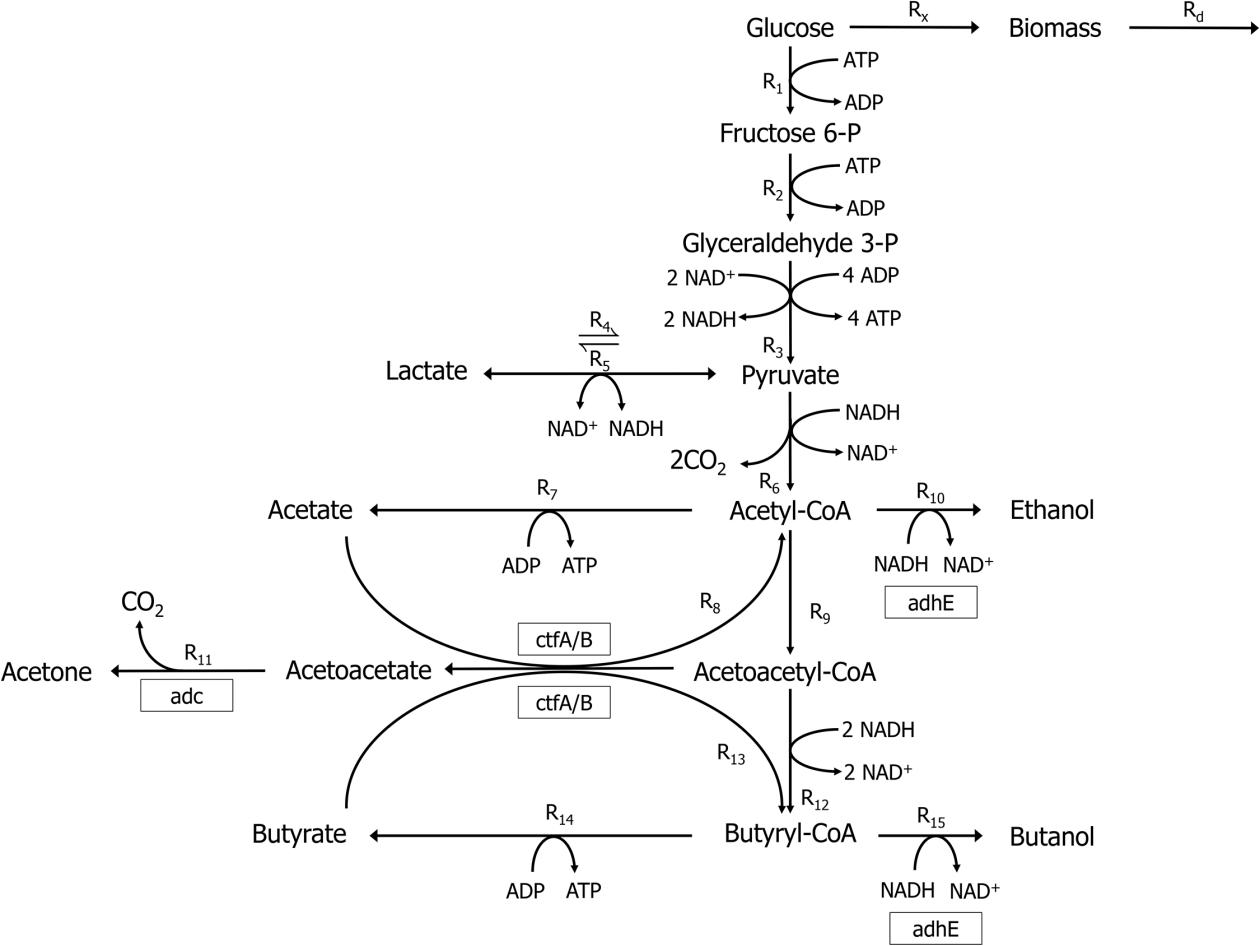
The metabolic pathway of *Clostridium acetobutylicum* (adopted from [25]).

The metabolic pathway of *C. acetobutylicum* is shown in Fig 1. The intermediates that have not been included in the presented kinetic model are omitted for clarity. Initially, the substrate glucose is converted to acetate and butyrate as the metabolism undergoes acidogenesis. Due to generation of the acidic metabolites, the external pH of the culture decreases until the cellular metabolism shifts to solventogenesis, a phase in which the acids are assimilated into acetone, butanol, and ethanol. Although this shift is associated with an external pH drop, its exact mechanism is not fully understood [45, 46]. As solventogenesis proceeds, the solvents eventually become toxic to *C. acetobutylicum*. In particular, butanol begins to disrupt the cellular membrane fluidity at concentration levels 8–10 g/L [14, 47]. The toxicity of butanol to *C. acetobutylicum* poses a key challenge since it limits the product titer. In fact, all the major products in the pathway—acetate, butyrate, acetone, butanol, and ethanol—are toxic to *C. acetobutylicum*, but only butanol and butyrate are typically at high enough concentration levels throughout the fermentation to cause substantial inhibition [14]. Running the ABE fermentation in a continuous culture helps alleviate product inhibition since the products can be continually removed from the culture. Product removal enables achieving higher product titers since the reactions are shifted toward the products.

In the continuous ABE fermentation, the shift to solventogenesis can be induced by changing the external pH from a high (approximately 5.5–6.0) to a low (approximately 4.5) level. Although over 100 different genes are upregulated after the switch to solventogenesis, the primary enzymes relevant to solventogenesis are encoded by the genes acetoacetate decarboxylase (adc), alcohol/aldehyde dehydrogenase (adhE), and butyrate-acetoacetate CoA-transferase (ctfA/B) [10]. These enzymes are responsive to the pH of the culture and change their expression accordingly. At acidogenic pH levels, the enzymes are present at a low concentration level. The enzymes are expressed more strongly when the fermentation switches to solventogenesis.

As the solvent production is dependent on the concentration of undissociated acids in the culture, it is often desirable to initially perform the continuous fermentation in the acidogenic stage so that sufficient butyrate is produced [8, 48]. Biomass growth is also impacted by the phase of the metabolism. During acidogenesis, *C. acetobutylicum* grows exponentially. As the metabolism shifts to solventogenesis, *C. acetobutylicum* begins to sporulate and reaches a stationary phase. Because the metabolic shift can be induced by changing the external pH, one common approach to avoid washout of biomass is to have a two-stage chemostat in which the first stage is operated at an acidogenic pH level and the second stage at a solventogenic pH level [8, 44, 49–51]. Growth during both phases is inhibited by the toxic acids and solvents produced during the fermentation. In addition to disrupting the cell membrane, butanol decreases the glucose uptake by *C. acetobutylicum* [14]. Ballongue et al. [52] have reported that the biomass growth rate decreases by 50% when either acetate or butyrate reaches 4 g/L, and the biomass growth stops when the total concentration of acids reaches approximately 5 g/L. Since butyrate is typically at a higher concentration level than acetate, it has a more deleterious effect on biomass concentration.

It has been shown that glucose inhibition also has a significant effect on the metabolism of *C. acetobutylicum*. Some of metabolic reactions that require an energy source such as ATP, which is correlated with the glucose concentration, are likely to shut off when there is a pronounced glucose inhibition [53]. The inclusion of certain intermediates such as pyruvate is critical for describing the cellular dynamics during glucose inhibition since these intermediates are affected by glucose inhibition.

### Modeling framework

#### Mass balance equations and reaction rates

Under the assumptions that the continuous *C. acetobutylicum* culture has a constant volume and temperature, the kinetic model of the metabolic pathway depicted in Fig 1 consists of (dynamic) mass balance equations for all metabolites in the pathway. The metabolites’ mass balance equations are listed in Table 1; reaction rates are labeled according to Fig 1. Glucose is the only metabolite that is fed to the culture. Hence, the dilution rate *D*, the inlet glucose concentration [G_in_], and the pH of the culture comprise the (adjustable) inputs to the continuous culture.

**Table 1:** The mass balance equations for all metabolites in the continuous *C. acetobutylicum* culture (see Fig 1). In all equations, [ ] denotes metabolite concentration (mM), R refers to rate equations (mM h^−1^), r is the base enzyme production rate during acidogenesis (mM h^−1^), r^+^ is the upregulated enzyme production rate during solventogenesis (mM h^−1^), H is defined in Eq (3), *D* is the dilution rate (h^−1^), and [G_in_] is the inlet glucose concentration (mM). The rate equations are given in Table 2.

$\text{Glucose:}\;\frac{d[\text{G}]}{dt}=-R_{1}-R_{x}-D\left(\left[\text{G}\right]-\left[{\text{G}}_{\text{in}}\right]\right)$	$\text{Acetate:}\;\frac{d[\text{A}]}{dt}=R_{7}-R_{8}-D\left[\text{A}\right]$
$\text{Fructose}\;\text{6-Phosphate:}\;\frac{d[{\text{F}}_{\text{6P}}]}{dt}=R_{1}-R_{2}-D\left[{\text{F}}_{\text{6P}}\right]$	$\text{Ethanol:}\;\frac{d[\text{En}]}{dt}=R_{10}-D\left[\text{En}\right]$
$\text{Glucose}\;\text{3-Phosphate:}\;\frac{d[{\text{G}}_{\text{3P}}]}{dt}=R_{2}-R_{3}-D\left[{\text{G}}_{\text{3P}}\right]$	$\text{Acetoacetyl-CoA:}\;\frac{d[\text{AaC}]}{dt}=R_{9}-R_{8}-R_{12}-R_{13}-D\left[\text{AaC}\right]$
$\text{Pyruvate:}\;\frac{d[\text{Py}]}{dt}=R_{3}+R_{4}-R_{5}-R_{6}-D\left[\text{Py}\right]$	$\text{Acetoacetate:}\;\frac{d[\text{Aa}]}{dt}=R_{8}+R_{13}-R_{11}-D\left[\text{Aa}\right]$
$\text{Lactate:}\;\frac{d[\text{Lac}]}{dt}=R_{5}-R_{4}-D\left[\text{Lac}\right]$	$\text{Butyryl-CoA:}\;\frac{d[\text{BC}]}{dt}=R_{12}+R_{13}-R_{14}-R_{15}-D\left[\text{BC}\right]$
$\text{Butyrate:}\;\frac{d[\text{B}]}{dt}=R_{14}-R_{13}-D\left[\text{B}\right]$	$\text{Acetyl-CoA:}\;\frac{d[\text{AC}]}{dt}=R_{6}+R_{8}-R_{7}-R_{9}-R_{10}-D\left[\text{AC}\right]$
$\text{Biomass:}\;\frac{d[\text{X}]}{dt}=R_{x}-R_{d}-D\left[\text{X}\right]$	$\text{Acetone:}\;\frac{d[\text{An}]}{dt}=R_{11}-D\left[\text{An}\right]$
$\text{Carbon}\;\text{Dioxide:}\;\frac{d[{\text{CO}}_{2}]}{dt}=R_{6}+R_{11}-D\left[{\text{CO}}_{2}\right]$	$\text{Butanol:}\;\frac{d[\text{Bn}]}{dt}=R_{15}-D\left[\text{Bn}\right]$
$\text{Adc:}\;\frac{d[\text{Ad}]}{dt}=r_{\text{Ad}}+r_{\text{Ad}}^{+}\text{H}-D\left[\text{Ad}\right]$	$\text{CtfA/B:}\;\frac{d[\text{Cf}]}{dt}=r_{\text{Cf}}+r_{\text{Cf}}^{+}\text{H}-D\left[\text{Cf}\right]$
$\text{AdhE:}\;\frac{d[\text{Ah}]}{dt}=r_{\text{Ah}}+r_{\text{Ah}}^{+}\text{H}-D\left[\text{Ah}\right]$	

The kinetic rate expressions for all the metabolic reactions are given in Table 2 with the kinetic parameters being listed in Table 3 and S1 Supporting Information. Most of the reactions in the metabolic pathway are represented by a typical enzyme-substrate reaction scheme

$$\begin{array}{c} E+S\underset{k_{-1}}{\overset{k_{1}}{⇌}}ES\overset{k_{2}}{\to }E+P. \end{array}$$ (1)

The rate expressions for these reactions are described by the Michaelis-Menten kinetics [54]

$$\begin{array}{c} \frac{d[\text{P}]}{dt}=\frac{V_{max}\left[\text{S}\right]}{K_{S}+\left[\text{S}\right]}, \end{array}$$

where [S] and [P] denote the concentration of substrate *S* and product *P*, respectively (mM); *V*_*max*_ is the maximum reaction rate (h^−1^); and *K*_*S*_ is the Michaelis constant (mM). Biomass is modeled as a substrate in the kinetic rates since all the metabolic reactions depend on the concentration of biomass (see Table 2). To describe the effect of glucose inhibition on the pathway dynamics, an on-off switch (denoted by F in the kinetic rates) is applied to reactions that utilize glucose as their energy source. These reactions involve the energy sources ATP, ADP, NADH, or NAD^+^, the availability of which is dependent on the glucose concentration [25]. The on-off switch describing the glucose inhibition effect is defined by the piecewise constant function [36]

$$\begin{array}{c} \text{F}=\{\begin{array}{c} 1,\;[\text{G}]>2\;\;\text{mM}\\ 0,\;[\text{G}]\leq 2\;\;\text{mM}, \end{array} \end{array}$$ (2)

where [G] denotes the concentration of glucose.

**Table 2:** The reaction rates in the metabolites’ mass balance equations listed in Table 1. F describes the effect of glucose inhibition (see Eq (2)). In all equations, *V* is the maximum reaction rate (mM h^−1^), *K* is the Michaelis constant (mM), *α* is the kinetic parameter described by Eq (4), *k*_d_ is the first order biomass death constant (h^−1^), and *μ* is the specific biomass growth rate (h^−1^).

$R_{1}=\frac{2(V_{1}\left[\text{G}\right]\left[\text{X}\right])}{K_{1}+\left[\text{G}\right]}\text{F}$	$R_{7}=\frac{V_{7}\left[\text{AC}\right]\left[\text{X}\right]}{K_{7}+\left[\text{AC}\right]}\text{F}$	*R*_13_ = *α*_13_[B][AaC][Cf][X]
$R_{2}=\frac{V_{2}\left[{\text{F}}_{\text{6P}}\right]\left[\text{X}\right]}{K_{2}+\left[{\text{F}}_{\text{6P}}\right]}\text{F}$	*R*_8_ = *α*_8_[A][AaC][Cf][X]	$R_{14}=\frac{V_{14}\left[\text{BC}\right]\left[\text{X}\right]}{K_{14}+\left[\text{BC}\right]}\text{F}$
$R_{3}=\frac{V_{3}\left[{\text{G}}_{\text{3P}}\right]\left[\text{X}\right]}{K_{3}+\left[{\text{G}}_{\text{3P}}\right]}\text{F}$	$R_{9}=\frac{V_{9}\left[\text{AC}\right]\left[\text{X}\right]}{2(K_{9}+\left[\text{AC}\right])}$	*R*_15_ = *α*_15_[BC][Ah][X]F
$R_{4}=\frac{V_{4}\left[\text{Lac}\right]\left[\text{X}\right]}{K_{4}+\left[\text{Lac}\right]}\text{F}$	*R*_10_ = *α*_10_[AC][Ah][X]F	*R*_d_ = *k*_*d*_[X]
$R_{5}=\frac{V_{5}\left[\text{Py}\right]\left[\text{X}\right]}{K_{5}+\left[\text{Py}\right]}\text{F}$	*R*_11_ = *α*_16_[Aa][Ad][X]	$R_{\text{x}}=\frac{\mu [\text{G}][\text{X}]}{K_{X}+\left[\text{G}\right]}$
$R_{6}=\frac{V_{6}\left[\text{Py}\right]\left[\text{X}\right]}{K_{6}+\left[\text{Py}\right]}\text{F}$	$R_{12}=\frac{V_{12}\left[\text{AaC}\right]\left[\text{X}\right]}{K_{12}+\left[\text{AaC}\right]}\text{F}$	

**Table 3:** The estimated kinetic parameters and their 95% confidence intervals.

Parameter	Value
*K*_1_	18.7 ± 0.410 mM
*K*_6_	0.00350 ± 9.54 × 10^−6^ mM
*K*_7_	0.0655 ± 0.0030 mM
*K*_9_	1.15 × 10^4^ ± 29.3 mM
*K*_*i*_	1340 ± 188 mM
*V*_1_	1.61 ± 0.0036 h^−1^
*V*_9_	4.91 × 10^6^ ± 1.25 × 10^4^ h^−1^
*α*_8_	4.53 × 10^3^ ± 43.4 mM^−2^h^−1^
*α*_10_	0.0761 ± 0.0198 mM^−2^h^−1^
*μ*_max_	0.126 ± 6.98 × 10^−4^ h^−1^
$r_{Ah}^{+}$	10.9 ± 0.758 mM h^−1^

The reaction rate expressions that have not been described by the Michaelis-Menten kinetics include the rates of biomass growth (*R*_X_) and death (*R*_d_) as well as the rates of enzyme regulation reactions (*R*_8_, *R*_10_, *R*_11_, *R*_13_, and *R*_15_). The rate expressions for these reactions are discussed below.

#### Biomass growth and death

The dynamics of the biomass concentration in the continuous *C. acetobutylicum* culture are governed by the biomass growth rate *R*_*X*_ and death rate *R*_*d*_. The biomass death kinetics are described by a first order expression [36]. On the other hand, biomass growth is dependent on the metabolic phase of the fermentation (and, consequently, on the culture pH) as well as the concentration of the inhibitory metabolites. The growth of biomass is generally inhibited by acetate, butyrate, acetone, butanol, and ethanol since all these metabolites are toxic to the growth of *C. acetobutylicum*. However, only butyrate and butanol typically reach the toxic levels that inhibit the biomass growth [14].

In this work, the biomass growth is described by the Monod kinetics expression [55]

$$\begin{array}{c} R_{\text{X}}=\frac{\mu [\text{G}][\text{X}]}{K_{\text{X}}+\left[\text{G}\right]}, \end{array}$$

where the observed specific growth rate of the biomass, *μ*, is defined by

$$\begin{array}{c} \mu =\mu_{\text{max}}F_{\text{T}}([\text{Bn}],[\text{B}],\text{pH}) \end{array}$$

with *μ*_max_ being the maximum specific growth rate and $F_{\text{T}}([\text{Bn}],[\text{B}],\text{pH})$ being the total inhibition function that accounts for the inhibitory effects of butanol, butyrate, and the culture pH on the biomass growth. The total inhibition function $F_{\text{T}}$ is defined by

$$\begin{array}{c} F_{\text{T}}([\text{Bn}],[\text{B}],\text{pH})=F_{\text{Bn}}F_{\text{B}}F_{\text{pH}}, \end{array}$$

where $F_{\text{Bn}}$, $F_{\text{B}}$, and $F_{\text{pH}}$ denote the inhibition functions for the butanol, butyrate, and pH effects, respectively.

Butanol inhibits the biomass uptake of glucose with noncompetitive inhibition kinetics [56]. Thus, the butanol inhibition function takes the form

$$\begin{array}{c} F_{\text{Bn}}([\text{Bn}])=\frac{1}{1+\frac{\left[\text{Bn}\right]}{K_{i}}}, \end{array}$$

where [Bn] is the concentration of butanol and *K*_*i*_ is an inhibition constant (mM). The butyrate inhibition function is given by [57]

$$\begin{array}{c} F_{\text{B}}([\text{B}])=1-{\left(\frac{\left[\text{B}\right]}{\left[{\text{B}}_{\text{max}}\right]}\right)}^{m_{b}}, \end{array}$$

where [B] is the concentration of butyrate, [B_max_] = 125.0 mM is the toxic concentration of butyrate, and *m*_*b*_ is a constant.

Biomass growth is dependent on the metabolic phase, with exponential growth during acidogenesis and stationary growth during solventogenesis. The metabolic phase of the continuous fermentation is affected by the external pH. Since the biomass growth is high during acidogenesis, high (acidogenic) pH does not inhibit the biomass growth, whereas low (solventogenic) pH does inhibit growth. Thus, the pH inhibition function is defined by [57]

$$\begin{array}{c} F_{\text{pH}}(\text{pH})=\{\begin{array}{cc} 1-m_{p}\left(5.6-\text{pH}\right),\; & \text{pH}\leq 5.6\\ 1,\; & \text{pH}>5.6, \end{array} \end{array}$$

where *m*_*p*_ is a constant. The pH inhibition function indicates that the biomass exhibits the highest growth rates at pH 5.6 (and higher pH levels) and less growth at lower pH levels.

#### Enzyme regulation

Since the upregulation of the enzymes adc, adhE, and ctfA/B is dependent on pH, the metabolic reactions involving these enzymes cannot be described by the Michaelis-Menten kinetics. Following the approach presented in [40], it is assumed that the total enzyme concentration in the culture does not remain constant. The mass balance for each enzyme is written as

$$\begin{array}{cc} \frac{d[\text{E}]}{dt} & =r_{E}+r_{E}^{+}\text{H}\;-\;D\left[\text{E}\right], \end{array}$$

where [E] denotes the concentration of the enzyme (i.e., adc, adhE, or ctfA/B) (mM); *r*_*E*_ and $r_{E}^{+}$ are constants (mM h^−1^); and H is a smoothed switch function defined by

$$\begin{array}{c} \text{H}=1-\text{tanh}(5[\text{pH}-4.5]). \end{array}$$ (3)

In Eq (3), the value of 4.5 represents the pH at which the switch occurs and the constant 5 determines the sharpness of the switch function. At low pH levels (∼4.5) the switch has a value of 1 and at high pH levels (∼ 6.0) the switch has a value of 0.

The constant *r*_*E*_ corresponds to the base enzyme production rate when the enzyme is downregulated (i.e., at high pH during acidogenesis). After the switch to solventogenesis (low pH), the enzyme is upregulated and is produced at the higher rate $r_{E}^{+}$. Physically, these constants represent the rates at which the enzyme is produced during the downregulated phase (acidogenesis) and upregulated phase (solventogenesis), respectively. These production rates are added to the outlet dilution term to obtain the overall mass balance for each enzyme.

Once the concentration of the enzymes is determined, the kinetic rates for the metabolites created from the enzymatic reactions can be described by

$$\begin{array}{c} \frac{d[\text{P}]}{dt}=-\alpha \left[\text{S}\right]\left[\text{E}\right],\;\alpha =\frac{k_{1}k_{2}}{k_{-1}+k_{2}}, \end{array}$$ (4)

where [P], [S], and [E] denote the concentration of the product, substrate, and enzyme, respectively; and *k*_1_, *k*_−1_, and *k*_2_ (h^−1^) are defined as in the reaction scheme Eq (1). This expression is derived using the pseudo steady-state approximation for the enzyme-substrate reactions with variable enzyme concentration.

### Parameter estimation and uncertainty analysis

The kinetic parameters that arise in the rate equations listed in Table 2 are largely unknown a priori and, therefore, must be estimated from experimental data. The large number of metabolites in the metabolic pathway depicted in Fig 1 results in a large number of parameters in the presented kinetic model. These include the parameters *K* and *V* that appear in each reaction rate described by the Michaelis-Menten kinetics, the parameters *α* in the enzyme regulation kinetics, and the parameters of the biomass growth and death rates (i.e., *k*_*d*_, *k*_12_, *μ*_max_, *K*_*i*_, *m*_*b*_, and *m*_*p*_). On the other hand, there is scant experimental data available for most intermediates in the pathway. Only the time profiles for the acidic metabolites (acetate, butyrate), solvents (acetone, butanol, ethanol), glucose, and biomass are typically measured in the continuous ABE fermentation [8, 9, 12, 13, 44]. The lack of experimental data poses a key challenge to parameter estimation since overfitting a large number of unknown parameters based on limited observations of the metabolic pathway can lead to a kinetic model with poor predictive capability. Although additional metabolic intermediates such as acetaldehyde and butyraldehyde exist in the metabolic pathway of *C. acetobutylicum* [22, 58, 59], only those present in [36] were included in order to reduce overparameterization and overfitting of the model.

In this work, the measured concentration of acetate, acetone, butyrate, butanol, ethanol, and glucose, i.e.,

$$\begin{array}{c} y={\left[\text{[A]}\;\text{[An]}\;\text{[B]}\;\text{[Bn]}\;\text{[En]}\;\text{[G]}\right]}^{⊤}, \end{array}$$

reported in [44] is used to estimate the kinetic parameters

$$\begin{array}{c} \theta ={\left[K_{1}\;K_{6}\;K_{7}\;K_{9}\;K_{i}\;V_{1}\;V_{9}\;\alpha_{8}\;\alpha_{10}\;\mu_{\text{max}}\;\;r_{Ah}^{+}\right]}^{⊤} \end{array}$$

that most directly affect the measured metabolites. In [44], the ABE fermentation was performed under limited phosphate (0.7 mM) and excess nitrogen in a continuous *C. acetobutylicum* ATCC 824 culture at pH 4.5. The inlet glucose concentration and the dilution rate were set to 167 mM (30 g/L) and 0.06 h^−1^, respectively.

The parameter estimation is posed as a weighted nonlinear least-squares optimization problem [60]

$$\begin{array}{cc} \hat{\theta }≔\arg & \min_{\theta }\sum_{i=1}^{N_{m}}\sum_{j=1}^{N_{v}}{∥{\bar{y}}_{ij}-y_{ij}∥}_{\text{Q}}^{2}\\ & \text{s.t.:}\;\text{model}\;\text{equations}, \end{array}$$ (5)

where $\hat{\theta }$ denotes the estimated value of the kinetic parameters *θ*; $\bar{y}$ and *y* denote the measured and predicted concentration of the metabolites; *N*_*m*_ is the number of measured metabolites (*N*_*m*_ = 6); *N*_*v*_ is the number of measurement points (*N*_*v*_ = 31); and the subscript *ij* denotes the *j*^*th*^ value of the *i*^*th*^ measured metabolite. The diagonal weight matrix Q in Eq (5) is defined in terms of the inverse of the standard deviation of concentration measurement noise of the metabolites

$$\begin{array}{c} \text{Q}=\text{diag}(0.143,0.150,0.260,0.208,0.513,0.559). \end{array}$$

The objective of the parameter estimation problem Eq (5) is to determine the kinetic parameters *θ* by minimizing the weighted squared difference between the predicted and measured concentration of the metabolites in *y*. The nonlinear optimization problem is solved in MATLAB using the genetic algorithm routine ga from the Global Optimization Toolbox. The parameter bounds considered in the optimization problem are given in S2 Supporting Information.

The uncertainty associated with the estimated parameters $\hat{\theta }$ is characterized in terms of the parameter variance-covariance matrix $V_{\theta }$ [60]

$$\begin{array}{c} V_{\theta }={\left(\sum_{i=1}^{N_{m}}\sum_{j=1}^{N_{v}}Q_{i}^{2}(\frac{\partial y_{ij}}{\partial \theta }{|}_{\theta =\hat{\theta }}\right)}^{⊤}{\left(\frac{\partial y_{ij}}{\partial \theta }{|}_{\theta =\hat{\theta }})\right)}^{-1} \end{array}$$

with *Q*_*i*_ being the *i*^*th*^ diagonal entry of the weight matrix Q and $\frac{\partial y_{ij}}{\partial \theta }$ denoting the parameter sensitivities. The boundaries of the parameter uncertainty region correspond to contours of constant probability density. When projected onto the parameter space, the uncertainty region takes the form of the ellipsoidal contour

$$\begin{array}{c} {\left(\theta -\hat{\theta }\right)}^{⊤}V_{\theta }^{-1}\left(\theta -\hat{\theta }\right) \end{array}$$

that has a $\chi_{p}^{2}$ probability distribution with *N*_*P*_ degrees of freedom (*N*_*P*_ being the number of estimated parameters) [61]. This implies that the estimated parameters $\hat{\theta }$ lie in an ellipsoidal uncertainty region with a probability level greater than *α*, that is,

$$\begin{array}{c} \hat{\theta }\in \left\{\theta |{\left(\theta -\hat{\theta }\right)}^{⊤}V_{\theta }^{-1}\left(\theta -\hat{\theta }\right)\leq \chi_{p}^{2}\left(\alpha \right)\right\}. \end{array}$$ (6)

The uncertainty region Eq (6) allows for defining the 95% confidence intervals for the estimated parameters as

$$\begin{array}{c} \theta_{i}\approx {\hat{\theta }}_{i}\pm \sqrt{\chi_{p}^{2}\left(\alpha \right)V_{\theta }\left(i,i\right)}, \end{array}$$

where $V_{\theta }\left(i,i\right)$ denotes the diagonal entries of the parameter variance-covariance matrix $V_{\theta }$ and *α* = 0.025. In fact, the above expression describes the dimensions of a hyperbox in the parameter space that circumscribes the ellipsoidal confidence region [62].

Table 3 lists the estimated parameters obtained through the parameter estimation problem Eq (5) and the experimental data reported in [44]. The 95% confidence intervals of the estimated parameters are computed following the above uncertainty analysis procedure. The small confidence intervals for most of the parameters suggest that the uncertainty associated with the estimated parameters is reasonably small. This is attributed to the optimization-based parameter estimation approach adopted in this work that results in adequate estimates for the (unknown) true kinetic parameters. The predictive capability of the kinetic model equipped with the estimated parameters is examined in the next section.

## Results and Discussion

This section discusses the predictive capability of the kinetic model under various culture conditions. Existing experimental data is used to validate the steady-state predictions of the kinetic model under nominal culture conditions. Due to unavailability of experimental data for other culture conditions, the dynamic behavior of the kinetic model is evaluated qualitatively in terms of the known trends of the ABE fermentation in a continuous culture.

### Model predictions vs. experimental data under nominal culture conditions

**Fig 2:**
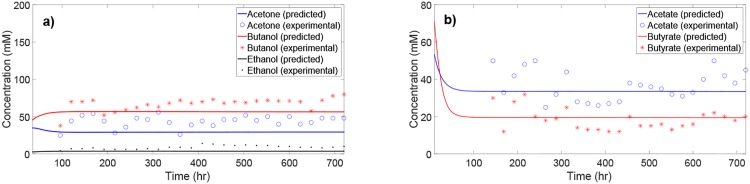
Comparison between model predictions and experimental time-course data (from [44]) for the key metabolites in continuous *C. acetobutylicum* culture.

The predictions of the kinetic model are validated against the experimental data from [44]. The chosen data set corresponds to the solventogenesis phase of the ABE fermentation in continuous *C. acetobutylicum* culture. The experimental data set includes the time-course concentration profiles for acetate and butyrate as well as the ABE solvents at a given dilution rate and culture pH. Fig 2 shows the predicted concentration profiles against the experimental data. The experimental concentration profiles are not shown for the initial phase of fermentation due to lack of reliable data. In [44], the experimental conditions and concentration of metabolites during the switch from the initial inoculated batch over to the continuous culture were not reported. Despite the noise in concentration measurements of the metabolites, Fig 2 suggests that the presented kinetic model can adequately predict the steady-state concentration of acetate and butyrate as well as that of the ABE solvents.

### Biomass pH dependence

**Fig 3:**
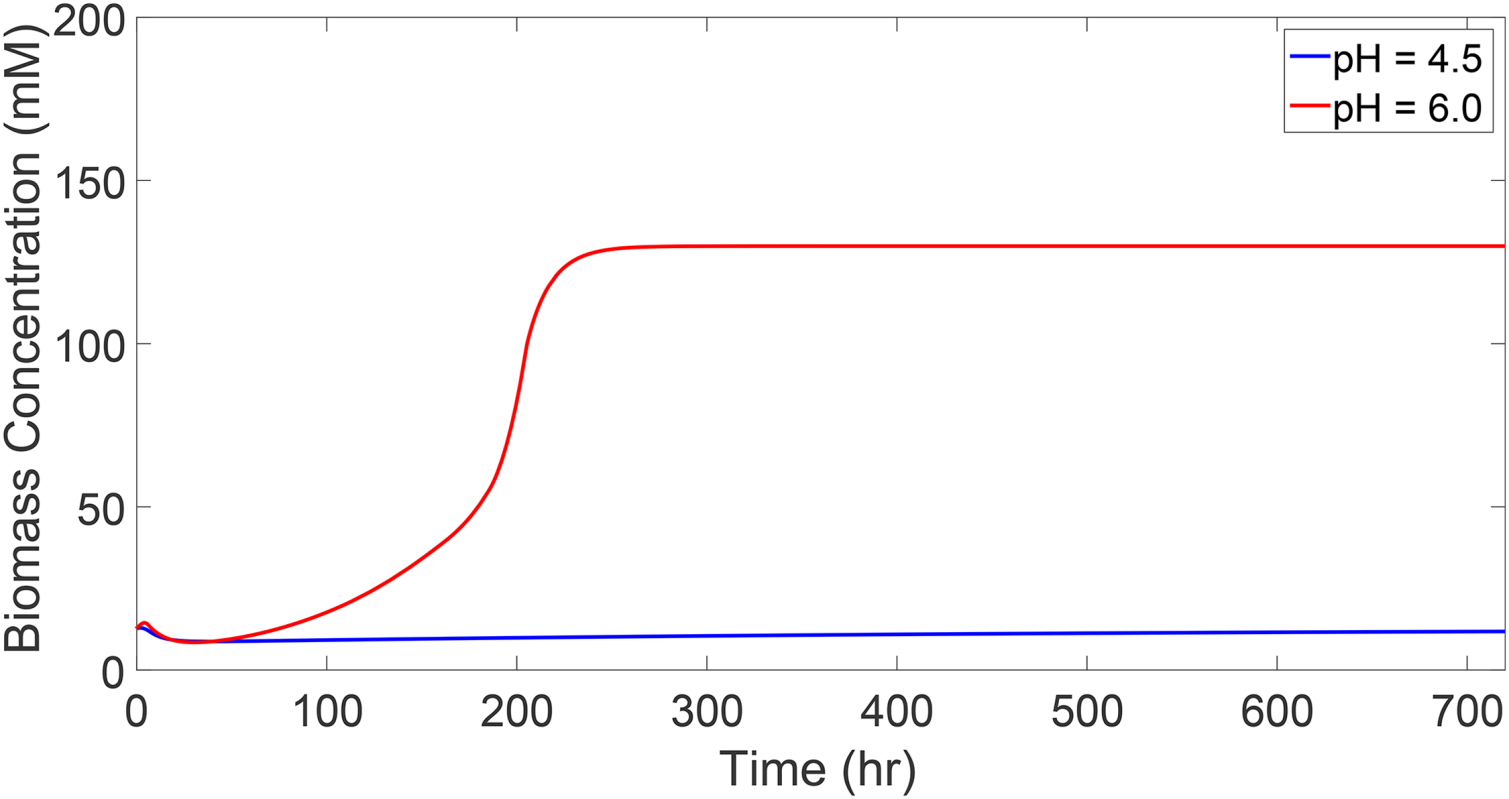
Biomass concentration profiles during the two metabolic phases of fermentation. The pH level 4.5 corresponds to the stationary biomass concentration during solventogenesis, while the pH level 6.0 corresponds to the exponential biomass growth during acidogenesis.

The pH of the culture affects the metabolic switch from acidogenesis to solventogenesis and, consequently, affects the biomass growth. Previous experimental work indicates that biomass growth tends to be maximum at the acidogenic pH levels, while the lower pH levels during solventogenesis inhibit biomass growth [8, 11, 48, 57]. This is due to the fact that *C. acetobutylicum* undergo exponential growth during acidogenesis, whereas they reach a stationary phase during solventogenesis. Fig 3 shows the model predictions at the pH levels 4.5 and 6.0 when other culture conditions are identical. As can be seen, at the solventogenic pH level 4.5, the biomass concentration is constant for essentially the entire fermentation time. At the pH level 6.0, however, the biomass grows exponentially before reaching steady state due to death and washout of *C. acetobutylicum*. This behavior is consistent with reported experimental results [12, 44, 63, 64].

### Product inhibition

**Fig 4:**
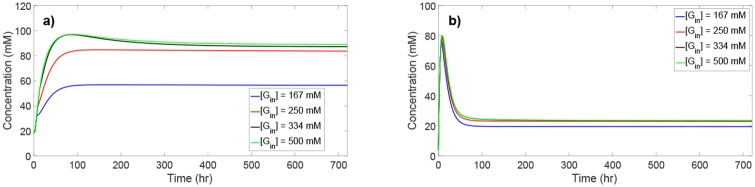
Butanol and butyrate concentration profiles for different inlet glucose concentration levels. Due to the product inhibition effect, the concentration of butanol and butyrate asymptotically reaches a maximum level as the inlet glucose concentration increases.

Butanol and butyrate are the primary inhibitory metabolites in the culture that hinder further biomass growth and solvent production. Even with abundant glucose present in the culture, *C. acetobutylicum* cannot indefinitely convert glucose since the increasing levels of butanol and butyrate will become toxic to *C. acetobutylicum* [14]. To demonstrate this effect, the inlet glucose concentration is increased from its nominal level in a stepwise manner. As the availability of glucose in the culture increases, the concentration of all metabolites is expected to increase when there is no product inhibition. Fig 4 shows the butanol and butyrate concentration profiles for four different inlet glucose concentrations. In the case of inlet glucose concentrations of 167 mM and 250 mM, *C. acetobutylicum* do not exhibit significant inhibition as evidenced by the increase in concentration of butanol and butyrate with the rising inlet glucose concentration levels. As the inlet glucose concentration increases further to 334 mM and 500 mM, the concentration of butanol and butyrate asymptotically reaches a maximum level of approximately 95 mM (7.0 g/L) and 80 mM (7.0 g/L), respectively. These concentration levels correspond to the toxic levels at which *C. acetobutylicum* undergo product inhibition, which hinders further glucose conversion [25, 28]. The higher levels of inlet glucose concentration will also cause glucose uptake inhibition. Thus, in practice, inhibition is due to the combined effects of both glucose uptake inhibition and product inhibition.

### The effect of pH on the metabolic switch

**Fig 5:**
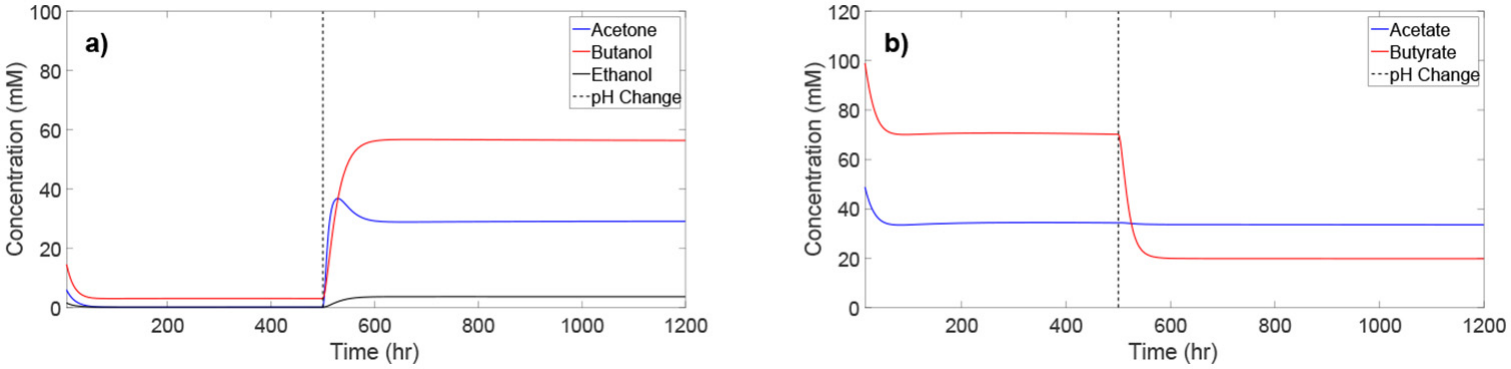
Concentration profiles for ABE solvents and acids at acidogenic pH (6.0) and solventogenic pH (4.5). At time 500 hr the culture pH is switched from 6.0 to 4.5 to demonstrate the shift from acidogenesis to solventogenesis.

The phase of the ABE fermentation (acidogenesis or solventogenesis) is strongly influenced by the culture pH. In the continuous ABE fermentation, the culture pH can be changed to induce a switch from one phase to another. To observe the switch between acidogenesis and solventogenesis, Fig 5 demonstrates the effect of changing the culture pH from an acidogenic pH level (6.0) to a solventogenic pH level (4.5) at time 500 hr. At high pH, acid production is high and solvent production is low. However, after the pH is lowered, the acids are assimilated into the solvents, and solvent production dramatically increases. This behavior indicates that the pH change from 6.0 to 4.5 triggers a switch from acidogenesis to solventogensis. One observation to note is that while the butyrate concentration decreases dramatically after the pH shift, the acetate concentration decreases only by a slight amount. This result is an indication that the nominal solventogenic culture conditions under which the kinetic model was validated (see Fig 2) have not been properly tuned for solvent production, as excess acetate remains present.

### Butanol productivity in relation to dilution rate and culture pH

**Fig 6:**
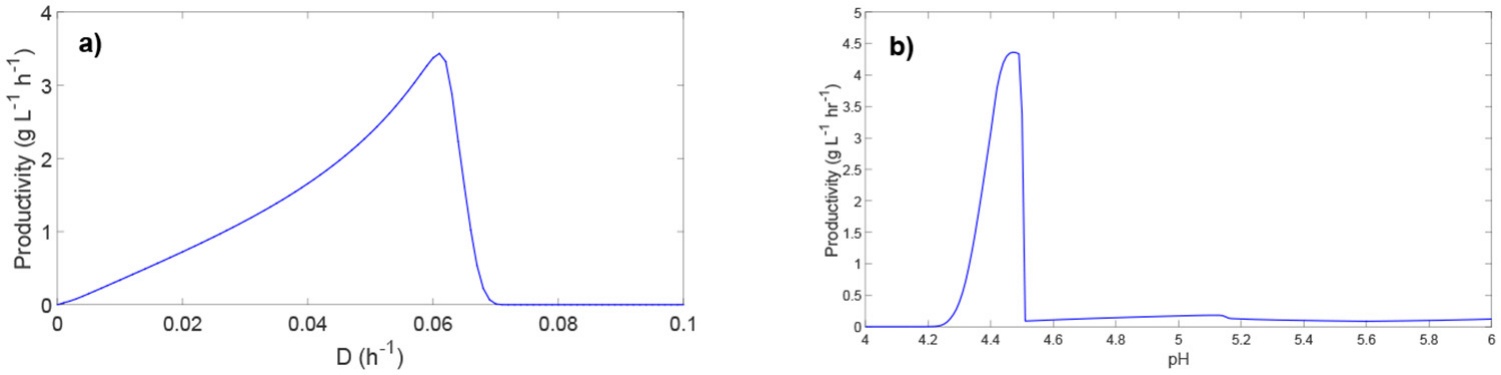
The effect of dilution rate and culture pH on steady-state butanol productivity.

The main culture (manipulated) inputs in the continuous ABE fermentation are the dilution rate and culture pH. Investigating butanol productivity in relation to these inputs can elucidate the culture conditions under which ABE fermentation is stable and butanol production is maximal. Typically, a high dilution rate is desired since higher dilution rates favor higher productivity. However, excessively high dilution rates can lead to biomass washout. To demonstrate the effect of dilution rate on the steady-state butanol productivity, the dilution rate was varied from 0 to 0.1 h^−1^ while holding pH at a constant level 4.5. Fig 6 shows that the steady-state butanol productivity increases with increasing dilution rate until *D* = 0.061 h^−1^, where a sharp drop to zero productivity is observed. This result suggests that the culture starts experiencing washout at this dilution rate.

Another input that can have a significant effect on the steady-state butanol productivity is the culture pH. Because the culture pH influences the metabolic phase of the fermentation, butanol productivity must be highest at low pH levels that correspond to the solventogenic phase. Fig 6 shows the steady-state butanol productivity for pH levels ranging from 4.0 to 6.0 at a constant dilution rate 0.06 h^−1^. The productivity is highest in the range of pH 4.3 to 4.7 with a peak value at approximately pH 4.45, suggesting that the fermentation is in the solventogenic phase in this pH range. Because biomass growth at pH levels lower than 4.3 becomes severely inhibited by the low pH [57], washout occurs at those conditions since the outlet dilution term dominates the biomass growth term of the mass balance. On the other hand, at the pH levels in the range of 4.6 to 6.0, the fermentation is in the acidogenic phase with very low butanol production. This behavior is consistent with the time-course data shown in Fig 5, which shows a high butanol concentration at pH 4.5 and a low butanol concentration at pH 6.0 due to the switch between the acidogenic and solventogenic phases. This is also consistent with previous experimental work [8, 11, 40, 44, 49] that has shown a culture pH of approximately 4.3–4.8 leads to the highest productivity and concentration of butanol [8, 11, 40, 44, 49].

**Fig 7:**
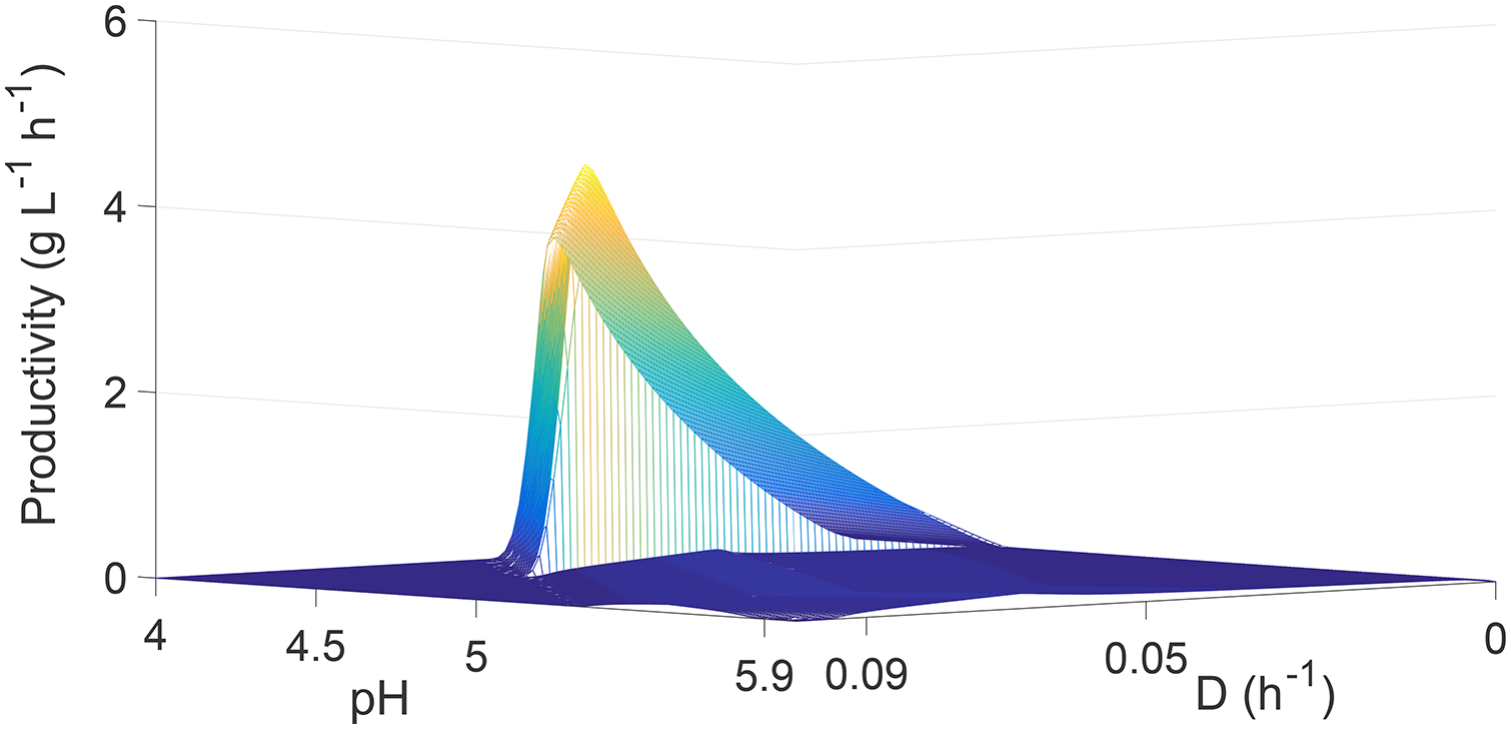
Steady-state butanol productivity as a function of dilution rate and culture pH.

To determine the culture conditions under which maximum butanol productivity is achieved, it is necessary to vary both dilution rate and pH simultaneously. Fig 7 shows the steady-state butanol productivity as a function of dilution rate (ranging from 0 to 0.1 h^−1^) and culture pH (ranging from 4 to 6). The maximum butanol productivity of 4.23 gL^−1^h^−1^ occurs at dilution rate and culture pH of 0.058 h^−1^ and 4.41, respectively. Notice that the maximum productivity occurs under different culture conditions in comparison with the results shown in Fig 6. The butanol productivity compares very favorably to the typical productivity of batch ABE fermentation (0.1–0.3 gL^−1^h^−1^), and is within the productivity range 0.4–6 gL^−1^h^−1^ previously reported for continuous ABE fermentation [5, 8, 65–67]. Fig 7 indicates the need for future model-based optimization studies for systematically identifying the optimal culture conditions that result in enhanced ABE production.

### Sensitivity of ABE concentration to enzyme production rates

**Fig 8:**
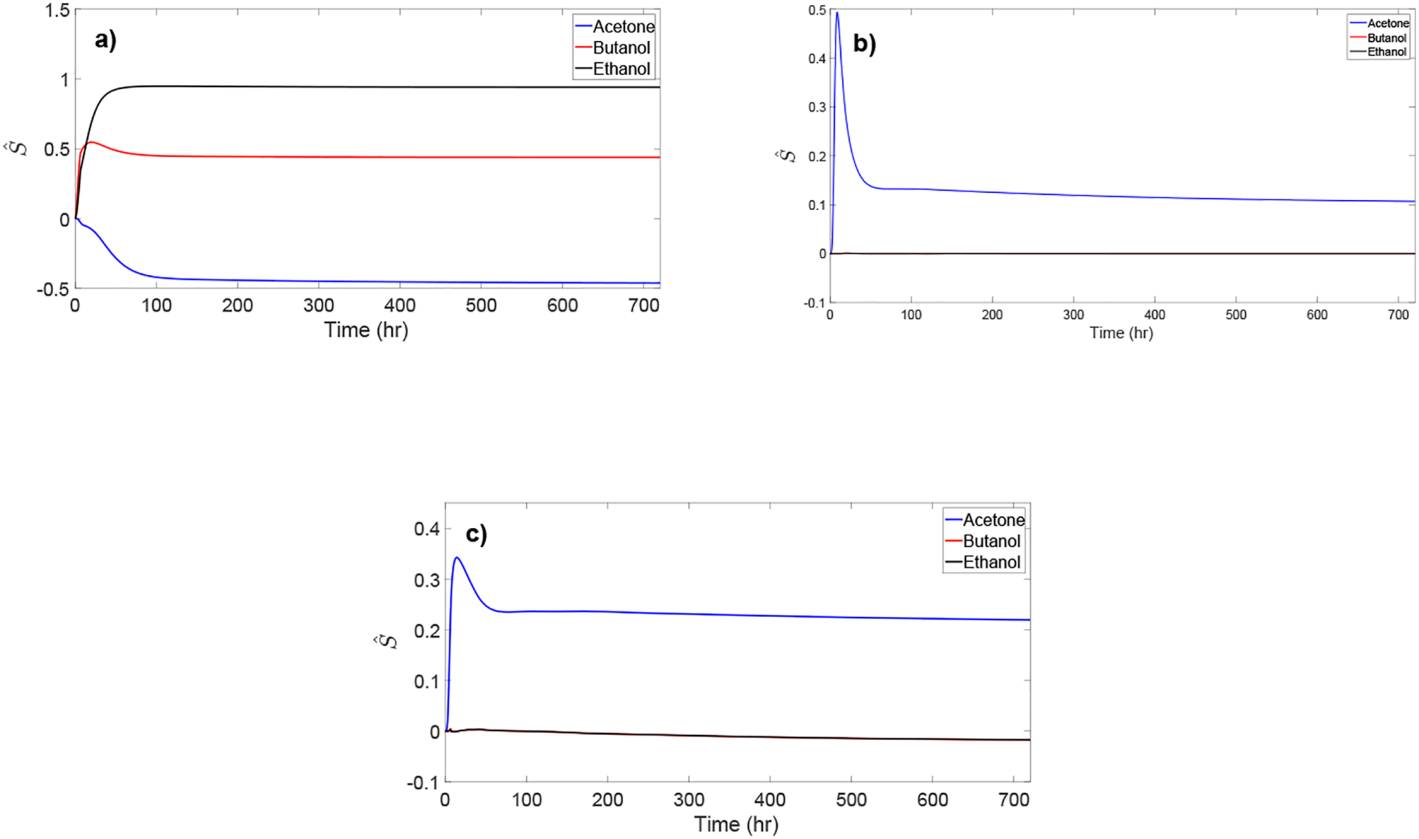
Normalized dynamic sensitivity of acetone, butanol, and ethanol concentration to the enzyme production rates.

To elucidate the effect of enzymes adhE, adc, and ctfA/B on concentration of the ABE solvents, a dynamic sensitivity analysis is performed with respect to the enzyme production rates $r_{\text{Ah}}^{+}$, $r_{\text{Ad}}^{+}$, and $r_{\text{Cf}}^{+}$. These enzyme kinetic parameters are selected due to their dominant effect on the enzyme production during solventogenesis. Dynamic sensitivity analysis allows for systematically investigating the effect of each enzyme production rate on concentration of the ABE solvents over the course of the fermentation. For each combination of the ABE solvent concentration *x* and kinetic parameter *p*, the normalized sensitivity is defined by

$$\begin{array}{c} \hat{S}=\frac{\partial x}{\partial p}\frac{\bar{p}}{\bar{x}}, \end{array}$$

where $\bar{p}$ and $\bar{x}$ denote the nominal values of *p* and *x*, respectively. Fig 8 shows the normalized dynamic sensitivity of concentration of the ABE solvents to the enzyme production rates. The production of adhE promotes higher concentration levels of butanol and ethanol but lower acetone concentration (see Fig 8). This is due to the fact that adhE is the enzyme responsible for the reactions that produce butanol and ethanol. Similarly, higher production of adc promotes higher acetone concentration with little effect on the concentration of butanol and ethanol since adc is only present in the metabolic reaction that produces acetone from acetoacetate. The enzyme CtfA/B is present in the backward reactions that consume acetate and butyrate (see Fig 1). Fig 8 shows that CtfA/B can have a large effect on the concentration of acetone while its effect on butanol and ethanol is insignificant. This implies that ctfA/B promotes the acetone production. Overall, Fig 8 suggests that, among the three enzymes, adhE is the strongest promoter of butanol and ethanol production, while acetone production is decreased with increased adhE production. Thus, engineering *C. acetobutylicum* to yield higher expression of adhE can possibly lead to higher butanol concentration and yield.

### Sensitivity of ABE concentration to reaction kinetics

**Fig 9:**
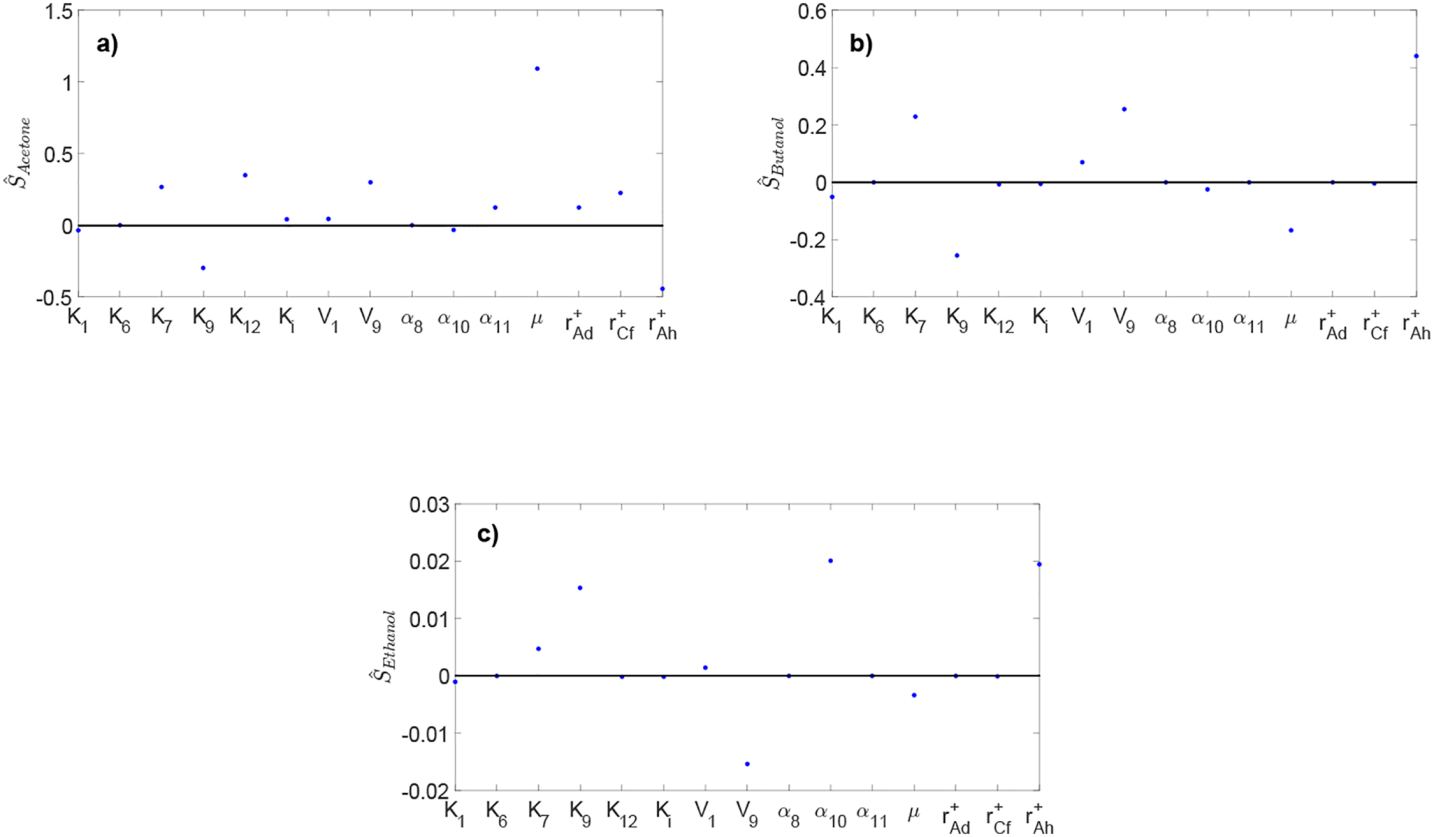
Normalized steady-state sensitivity of acetone, butanol, and ethanol concentration to different reaction kinetics along the metabolic pathway.

The normalized steady-state sensitivity of the ABE concentration to the kinetic parameters of different metabolic reactions along the pathway is shown in Fig 9. Acetone concentration exhibits the highest sensitivity to the kinetic parameters. On the other hand, ethanol concentration appears to be largely insensitive to the selected reaction kinetics. Butanol concentration is most sensitive to *K*_7_, *V*_9_, and $r_{\text{Ah}}^{+}$ with a positive effect, and most sensitive to *K*_9_ and *μ*_max_ with a negative effect. The high sensitivity of acetone and butanol concentration to *V*_9_ and *K*_9_ indicates that acetoacetyl-CoA (produced by reaction 9) is a key intermediate for the production of acetone and butanol; increasing the rate of acetoacetyl-CoA formation can promote higher acetone and butanol production. Fig 9 suggests that forming more acetate (through increasing *K*_7_) is likely to promote a higher acetone and butanol production. The rate of enzyme adhE production ($r_{\text{Ah}}^{+}$) and the maximum specific growth rate of biomass (*μ*_max_) have reverse effects on the concentration of acetone and butanol, while having little effect on the ethanol concentration. Hence, manipulating $r_{\text{Ah}}^{+}$ and *μ*_max_ can be an effective strategy for increasing the production selectivity toward butanol.

**Fig 10:**
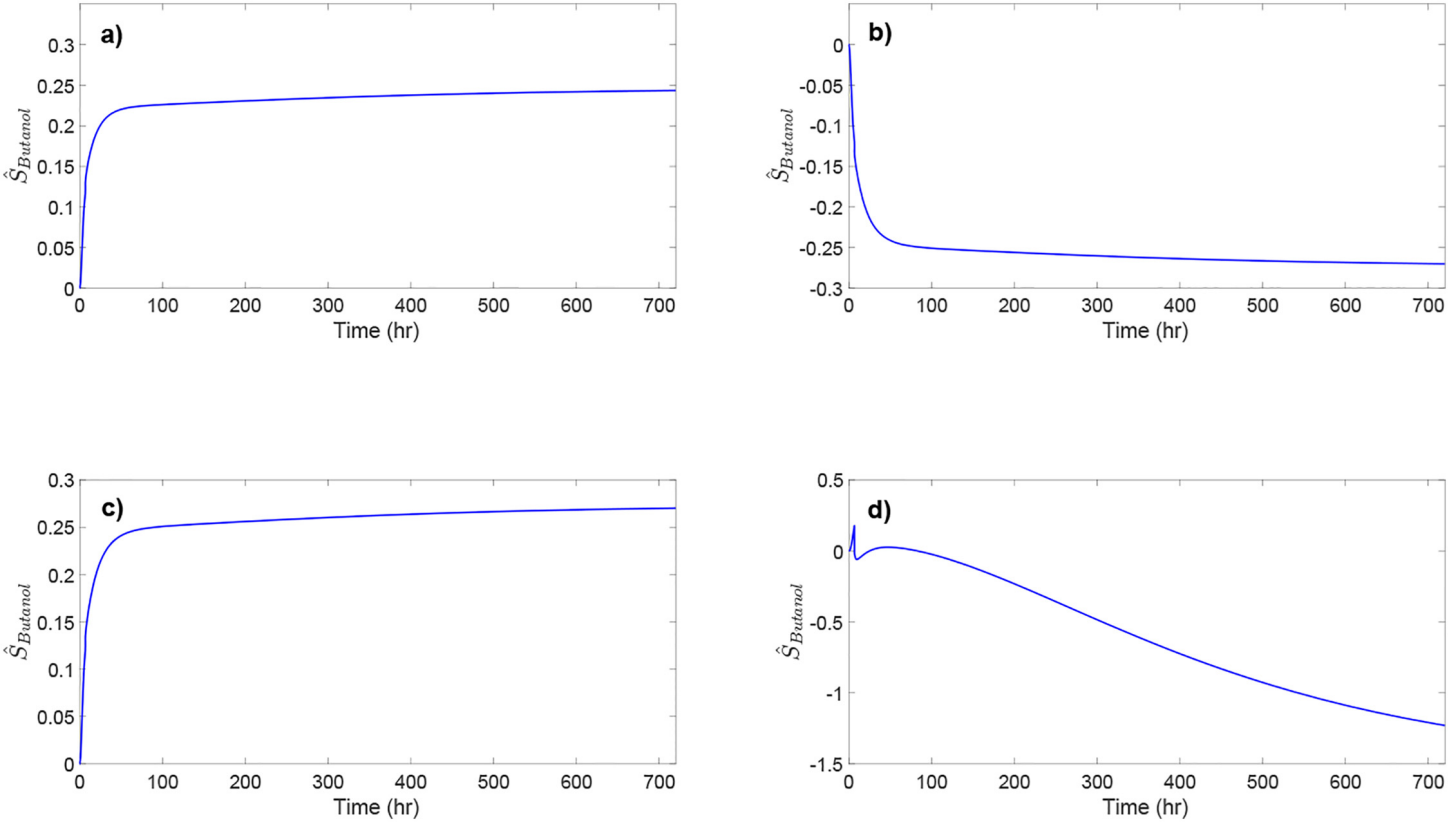
Normalized dynamic sensitivity of butanol concentration to the most sensitive reaction kinetics.

To further investigate the sensitivity of butanol production to the most sensitive reaction kinetics identified from the above analysis, the dynamic sensitivity of butanol concentration to *K*_7_, *K*_9_, *V*_9_, and *μ*_max_ is evaluated (see Fig 10); the sensitivity with respect to $r_{Ah}^{+}$ is shown in 8. Butanol concentration shows evolving sensitivity to these kinetic parameters over time. The magnitude of the sensitivity results is greater at steady state than that during the initial phase of the ABE fermentation. Fig 10 indicates the benefit of systems analysis of the metabolic pathway since the sensitivity of metabolite concentration to reaction kinetics may change dramatically during fermentation.

## Conclusions

A kinetic model has been presented for the ABE fermentation in continuous *Clostridium acetobutylicum* culture. The kinetic model includes the key intermediates and enzymes in the metabolic pathway, and describes the effects of culture pH, product inhibition, and glucose inhibition. The parameters of the kinetic model have been estimated in a weighted least-squares sense using literature data. The simulation results indicate that the presented kinetic model can adequately describe the trends of the ABE fermentation under various culture conditions as established in previous experimental studies. An extensive (dynamic) sensitivity analysis has been performed to elucidate the effect of metabolic reaction kinetics and enzyme production rates on the ABE production. In addition, the influence of culture pH and dilution rate on steady-state butanol productivity has been explored to determine the culture conditions that yield enhanced butanol production in a continuous culture. Systems analysis of the metabolic pathway sheds light onto identifying the key intermediates and enzymes for metabolic engineering as well as optimal design and operation of continuous ABE fermentation processes.

## Supporting Information

S1 Supporting Information Kinetic parameters of the reaction rates listed in Table 2. (PDF)

S2 Supporting Information The parameter bounds considered in the parameter estimation problem (5). (PDF)
