# Supplementary material for: Kinetic Study of Acetone-Butanol-Ethanol Fermentation in Continuous Culture
Source: PLoS One. 2016 Aug 3;11(8):e0158243. doi: 10.1371/journal.pone.0158243 (PMC4972440; doi:10.1371/journal.pone.0158243)
Supplement: S1 Supporting Information — (PDF) [file pone.0158243.s001.pdf]

Supporting Information A

Kinetic parameters of the reaction rates listed in Table 2.

Parameter	Value
K_1	18.7 mM
K_2	11.8 mM
K_3	10.0 mM
K_4	3.08 mM
K_5	1.00 mM
K_6	0.00350 mM
K_7	0.0655 mM
K_9	1.15 $\times 10^4$ mM
K_{12}	1.40 $\times 10^{-4}$ mM
K_{14}	1.00 $\times 10^{-6}$ mM
K_i	1340 mM
K_X	0.177 mM
V_1	1.61 h^{-1}
V_2	25.5 h^{-1}
V_3	100 h^{-1}
V_4	23.2 h^{-1}
V_5	1.00 h^{-1}
V_6	2.18 h^{-1}
V_7	8.80 h^{-1}
V_9	9.82 $\times 10^6$ h^{-1}
V_{12}	475 h^{-1}
V_{14}	600 h^{-1}
α_8	4.53 $\times 10^3$ mM^{-2} h^{-1}
α_{10}	0.0761 mM^{-2} h^{-1}
α_{11}	1.35 $\times 10^{-4}$ mM^{-2} h^{-1}
α_{13}	5.00 $\times 10^6$ mM^{-2} h^{-1}
α_{15}	3.47 $\times 10^6$ mM^{-2} h^{-1}
μ_{max}	0.126 h^{-1}
r_{Ad}	5.47 $\times 10^{-3}$ mM h^{-1}
r_{Cf}	3.24 $\times 10^{-4}$ mM h^{-1}
r_{Ah}	0.289 mM h^{-1}
r^+_{Ad}	19.3 mM h^{-1}
r^+_{Cf}	5.78 $\times 10^{-4}$ mM h^{-1}
r^+_{Ah}	10.9 mM h^{-1}
