# Supplementary material for: Kinetic Study of Acetone-Butanol-Ethanol Fermentation in Continuous Culture
Source: PLoS One. 2016 Aug 3;11(8):e0158243. doi: 10.1371/journal.pone.0158243 (PMC4972440; doi:10.1371/journal.pone.0158243)
Supplement: S2 Supporting Information — (PDF) [file pone.0158243.s002.pdf]

Supporting Information B

The parameter bounds considered in the parameter estimation problem (5).

Parameter	Bound
K_1	$[10^{-6}, 10^{2}]$ mM
K_6	$[10^{-6}, 10^{2}]$ mM
K_7	$[10^{-6}, 10^{2}]$ mM
K_9	$[1, 2 \times 10^{5}]$ mM
K_i	$[10^{-2}, 10^{4}]$ mM
V_1	$[10^{-2}, 10^{5}]$ h^{-1}
V_9	$[10^{-2}, 10^{5}]$ h^{-1}
α_8	$[10^{-2}, 10^{5}]$ $mM^{-2}h^{-1}$
α_{10}	$[10^{-2}, 10^{5}]$ $mM^{-2}h^{-1}$
$\mu_{\max}$	$[0.05, 0.8]$ h^{-1}
r^{+}_{Ah}	$[10^{-2}, 50]$ mM h^{-1}
